# Melkersson-rosenthal syndrome associated with 21 trisomy as a differential diagnosis of angioedema

**DOI:** 10.1186/1939-4551-8-S1-A98

**Published:** 2015-04-08

**Authors:** Mariana Monteiro, Estela Risso, Cintia Bassani, Catarina Furlan, Maria Elisa Andrade, Romero Kopke, João Ferreira De Mello, Erica Sbrissa

**Affiliations:** 1 Hospital Do Servidor Público Estadual De São Paulo, Brazil

## Background

The Melkersson-Rosenthal Syndrome (MRS) is a rare disease that often affects young adults and is characterized by the presence of a painless orofacial edema, congenital fissured tongue and recurrent unilateral or bilateral facial palsy. No predilection for sex or race. Although its etiology is unknown it is believed that genetic, vasomotor, infectious and allergic disorders may be associated. The diagnosis can be made by the association of symptoms with lip biopsy, which shows in the initial stage dilated lymphatics with aggregates of lymphocytes, histiocytes and plasma cells, and in the later stages there is granulomatous inflammation, no caseous with Langerhans giant cells. Due to the rarity of MRS, it should always be included in the differential diagnosis: inflammatory pseudotumor, allergic phenomena, Crohn's disease, hereditary angioedema, sarcoidosis, lymphangioma and hemangioma.

## Methods

Report a case of Melkersson-Rosenthal Syndrome associated with Trisomy 21 as a differential diagnosis of angioedema.

## Results

CAJ, 15 years old, female, carrier of Down Syndrome, came to the Allergy Department of the Hospital do Servidor Público Estadual in 2014. Presented facial paralysis on the left side in 2007 and on the right side in 2009, both with spontaneous reversion. In 2011, began presenting facial and lip edema, with progressive worsening. Reported a worsening with infectious conditions.

Physical examination: Labial angioedema 3 + / 4 +, fissured tongue and residual bilateral facial paralysis.

Laboratory tests: Infectious and Rheumatic: negatives.

Endocrine: Free T4 and Thyroglobulin Antibody: normal; Peroxidase Antibody and TSH both enhanced.

Immune: C3, C4 and Immunoglobulins: unchanged.

Biopsy of Upper Lip (06/30/2011): Fragment of labial mucosa with hyperkeratosis and acanthosis of the epithelium, vascular ectasia and inflammatory infiltrate in the corium. (04/30/2014): Malpighian mucosa with mild acanthosis in addition to mild superficial perivascular lymphocytic infiltrate deep. Note: the absence of granulomas.

## Conclusions

The diagnosis was confirmed by biopsy in a teenager with Down syndrome. This is a rare event in children, and very rarely associated with Down Syndrome. Until now, only 2 studies reported the association of MRS and Down Syndrome-however in adults. The combination of MRS and Hashimoto's thyroiditis is also unusual, with few cases described in the literature. The patient in this case is under investigation by having alteration of thyroid function.

## Consent

Written informed consent was obtained from the patient for publication of this abstract and any accompanying images. A copy of the written consent is available for review by the Editor of this journal.

